# Executive Functions Among Kindergarteners in the Capital of Laos: Relations with Socialization Practices and Later Mathematics Skills

**DOI:** 10.1111/desc.70302

**Published:** 2026-11-03

**Authors:** Yoko Kajiyama, Yusuke Takahashi, Yusuke Moriguchi

**Affiliations:** ^1^ Graduate School of Letters Kyoto University Kyoto Japan; ^2^ Graduate School of Education Kyoto University Kyoto Japan

**Keywords:** executive functions, early childhood, socialization, mathematics achievement, cultural values, social changes

## Abstract

Executive functions (EFs), conceptualized as conscious, goal‐directed control processes of thought, action, and emotion, develop rapidly during early childhood and predict various developmental outcomes. Previous research has linked socialization practices to early EFs and EFs to mathematics achievement, yet has largely focused on WEIRD (Western, Educated, Industrialized, Rich, and Democratic) contexts. This study examined EFs’ associations with socialization practices and later mathematics skills in the capital of Laos, a rapidly shifting sociodemographic context within a United Nations‐designated least‐developed, agricultural, and traditionally collectivistic country. In Study 1, 66 kindergarteners (*M_age_* = 54.8 months) performed EF and language tasks, and their caregivers reported on socialization practices (autonomy support and intrusive control). In Study 2, 35 children were followed up one year later to assess their mathematics performance. The results showed that kindergarteners’ EFs predicted later mathematics skills, providing additional evidence consistent with the possibility that this association extends across diverse sociodemographic contexts, while offering essential baseline data to support local mathematics education. In contrast, moderate evidence for a null association of intrusive control and anecdotal evidence for a negative association of autonomy support with children's EFs were found, consistent with the possibility that socialization‐EF relations may vary across sociocultural contexts rather than following universal patterns. Together, these findings suggest that EFs represent an important cognitive capacity for later mathematics achievement, tentatively highlighting the value of considering sociocultural contexts when modeling early socialization‐EF relations.

## Summary

- We examined kindergarteners’ executive functions (EFs) alongside socialization practices and later mathematics skills within a rapidly shifting sociodemographic context in Laos.
- Socialization‐EF associations were consistent with the possibility that these relationships may vary across sociocultural contexts.
- Early EFs predicted later mathematics achievement in kindergarteners, providing additional evidence consistent with the possibility that this association extends across diverse contexts.
- Findings provide a foundation for future research and may inform policy discussions on young children's cognitive and academic development across transitioning, non‐WEIRD, collectivistic contexts.

## 1 Introduction

Executive functions (EFs) are broadly conceptualized as higher‐order neurocognitive processes that support conscious, goal‐directed control of thought, action, and emotion. In developmental research, EFs are often distinguished into cool and hot components. Cool EFs refer to relatively abstract and decontextualized control processes, which often include inhibitory control, cognitive flexibility, and working memory, whereas hot EFs are engaged in affectively salient or motivationally charged situations (Moriguchi and Phillips 2023; Zelazo and Carlson 2012). EFs undergo rapid development during early childhood (Carlson 2005; Zelazo et al. 2003), in tandem with prefrontal cortex and fronto‐parietal control network maturation (Marek and Dosenbach 2018; Moriguchi and Hiraki 2013). While EFs are foundational for subsequent cognitive, social, and academic outcomes (e.g., Blair and Razza 2007; Devine and Hughes 2014; Moffitt et al. 2011), most evidence is derived from WEIRD (Western, Educated, Industrialized, Rich, and Democratic) contexts (Henrich et al. 2010; Moriguchi 2022). Consequently, it remains unclear to what extent core assumptions about EF development—particularly regarding its environmental determinants —hold in non‐WEIRD contexts.

Previous studies have examined both the environmental factors associated with young children's EFs and the developmental outcomes associated with individual differences in EFs. Among the various environmental factors, caregivers’ socialization practices have emerged as a particularly important correlate of early EF development (Fay‐Stammbach et al. 2014, for a review; Valcan et al. 2018, for a meta‐analytic review). In WEIRD contexts, autonomy support—characterized by taking children's perspective, respecting their rhythms, and fostering active task participation (Matte‐Gagné and Bernier 2011) — is consistently associated with advanced EFs (e.g., Bernier et al. 2010; Meuwissen and Carlson 2015; National Institute of Child Health and Human Development Early Child Care Research Network 2005). Conversely, caregivers’ intrusive control—defined as caregivers’ psychological or behavioral intrusiveness, pressure, or dominance (Grolnick and Pomerantz 2009; Pomerantz and Wang 2009) — frequently correlates with poorer EFs (e.g., Clark and Woodward 2015; Cuevas et al. 2014; Taylor et al. 2013).

Simultaneously, EFs predict diverse developmental outcomes, including theory of mind (Devine and Hughes 2014), academic achievement (Blair and Razza 2007), and physical, mental health, and long‐term socioeconomic outcomes (Moffitt et al. 2011). Within academics, EFs correlate strongly with general mathematics skills, such as calculation and reasoning, basic number knowledge, and spatial tasks (Emslander and Scherer 2022, for a systematic review and meta‐analysis). Moreover, preschool EFs robustly predict subsequent mathematics performance (e.g., Bull et al. 2008; Clark et al. 2010; McClelland et al. 2014; for reviews see Clements et al. 2016; Cragg and Gilmore 2014; Jacob and Parkinson 2015).

However, this paradigm is overwhelmingly based on WEIRD societies (Henrich et al. 2010; Moriguchi 2022). This limitation is especially problematic when considering the relationships between socialization practices and children's EFs because sociodemographic contexts like EIRD of WEIRD are conceptualized to link cultural values, socialization practices, and human development (Greenfield 2016 2018). According to this framework, macro‐level sociodemographic shifts from less to more EIRD environments (e.g., urbanization, commercialization, formal education, wealth, and smaller household sizes) are theorized to relate to shifts in cultural values from collectivistic (prioritizing obedience, respect, and hierarchical relations) to individualistic (prioritizing autonomy, uniqueness, and egalitarian relations). These shifting values are posited to associate with caregivers’ socialization goals and children's development. That is, as caregivers transition from emphasizing obedience to autonomy, their socialization practices may shift from intrusive control to autonomy‐support, which may be associated with differences in children's development. This theory raises questions about whether socialization practices identified as beneficial or detrimental to children's development in particular sociodemographic contexts (such as more‐EIRD contexts) function similarly in other sociodemographic contexts (such as less‐EIRD contexts), where cultural values may differ. Accordingly, there may be socioculturally different pathways towards important developmental goals such as EFs (Greenfield et al. 2003; regarding EFs: Cho et al. 2023; Lamm et al. 2018).

This argument is further supported by studies showing that socialization practices that appear similar on the surface may have different meanings and implications for both caregivers and children, leading to different associations with children's development in different cultures (Chao 1994; Cho et al. 2023; Tobin et al. 1989). Moreover, the more normative a socialization practice is within a given culture, the more positive/less negative its meanings and implications are for caregivers and children, leading to more positive/less negative associations with children's development (Lansford 2022; Lansford et al. 2005). In particular, the meanings and normativeness of caregivers’ autonomy support and intrusive control may differ across the spectrum of collectivist‐individualist cultural orientations. In more collectivistic cultures, intrusive control is often viewed as necessary for children's development and perceived by children as expressions of care and involvement, rather than coercion (Chao 1994; Cho et al. 2023; Tobin et al. 1989). In contrast, in such cultures, autonomy support is not particularly valued, and such cultural meanings and norms are internalized by children as well (Rudy and Grusec 2001). In fact, some studies show that socialization practices characterized by intrusive control have been shown to have barely negative, no, or even positive associations with children's development in more collectivistic cultures (Chao et al. 2001; Dwairy et al. 2006; Leung et al. 1998; Steinberg et al. 1994), unlike the negative associations shown in more individualistic cultures.

The recent dominant proposal is that while the positive associations of autonomy support and negative associations of intrusive control with children's development are universal, based on self‐determination theory (Deci and Ryan 1985), there is some adequate room for cultural specificities within the theory, accommodating culturally different meanings and norms of socialization practices (Cheung and Cheah 2025; Soenens et al. 2015; Pomerantz and Wang 2009). It is claimed that cultural similarities and specificities coexist in the relationships between socialization practices and children's development. This perspective is supported by some empirical findings (Asmara et al. 2025; Sun et al. 2025; Wang et al. 2007). For example, in a single study, Wang et al. (2007) found both positive associations of autonomy support and negative associations of intrusive control with child outcomes in both collectivistic and individualistic cultures, with the positive associations of autonomy support being weaker in the collectivistic culture.

Now, focusing on recent empirical research that investigated the relationships between socialization practices characterized by autonomy support and/or intrusive control and early EFs in contexts where the authors mention their collectivistic characteristics, both socialization practices have produced mixed results. Some studies showed associations similar to those observed in individualistic cultures, while others showed no associations. Intrusive control has been found to show negative (Zhai et al. 2023) or no association (Doan and Song 2022; Sun et al. 2025; Xie and Li 2022) with young children's EFs, while autonomy support has shown positive associations with the whole or only some aspects of EFs, and no associations with the others (Sun et al. 2025; Xie and Li 2022).

Given the mixed results, more studies are needed to investigate the associations between socialization practices (autonomy support and intrusive control) and early EFs in more diverse collectivistic cultural contexts. Therefore, the present study examined such associations in a traditionally collectivistic, highly underrepresented, and rapidly shifting sociodemographic context: the capital of the Lao People's Democratic Republic (Laos). Laos is scheduled to graduate from the United Nations‐designated least developed country status in 2026 (United Nations 2025), having sustained a 4–8% real GDP growth over the past two decades (United Nations Conference on Trade and Development 2025), and reduced its national poverty rate from 27.6% in 2007 to 15.0% in 2024 (Ministry of Finance and Lao Statistics Bureau 2025). Nevertheless, structural employment transition from low‐productivity agriculture to higher‐productivity non‐agricultural sectors has lagged, leaving over 70% of the population in agriculture (United Nations 2025; World Bank 2025). Traditionally, Laotian socialization reflects collectivistic values, with children raised by extended families and the broader community, and socialized to respect elders and fulfill social obligations (Kinslenco 2009; Ruiz‐Casares and Phommavong 2016). However, within Greenfield's (2016) framework, these values are theorized to align with broader social shifts, a process likely more pronounced in the capital than in other areas. Compared to national averages, capital residents are more likely to live in urban areas (77.9% vs. 32.9%; Lao Statistics Bureau 2015), escape poverty (an 8.3% poverty headcount rate vs. 15.0%; Ministry of Finance and Lao Statistics Bureau 2025), and be literate (97.2% of those aged 15 and older vs. 84.7%; Lao Statistics Bureau 2015). They also exhibit higher net school attendance ratios in early childhood, primary, lower secondary, and upper secondary education (63.8%, 95.0%, 82.9%, and 64.0%, respectively, vs. 32.1%, 89.6%, 60.5%, and 38.1% nationally; Lao Statistics Bureau 2018), alongside smaller households (4.6 vs. 5.3), and a lower crude birth rate (22 vs. 28) (Lao Statistics Bureau 2015). Therefore, this study in the Laotian capital examines the relationships between socialization practices and early EFs in a relatively more rapidly transitioning sociodemographic pocket inside a traditionally collectivistic and less‐EIRD (Greenfield 2018) context.

Turning to the longitudinal association between EFs and later mathematics skills—and in contrast to the potential cultural moderation of the socialization‐EF link—this association has generally been expected to be robust across contexts. Indeed, mathematical learning fundamentally relies on cognitive processes undergirded by EFs, including task goal maintenance, response inhibition, and flexible shifting between strategies (e.g., Emslander and Scherer 2022). Recent studies in non‐EIRD contexts have also supported an association between EFs and mathematics skills, including both longitudinal (Wolf and McCoy 2019) and cross‐sectional evidence (Finch et al. 2022). Empirically testing the boundary of this association within an understudied, transitional context helps advance developmental theories toward broader applicability (Cheung and Cheah 2025). Simultaneously, tracking this association addresses urgent national educational challenges. Nationally, Laotian children experience substantial difficulties in basic mathematics. While fewer than 1% of fifth‐graders achieve grade‐appropriate mathematics levels, 33% remain at an early primary level (United Nations International Children's Emergency Fund [UNICEF] and Southeast Asian Ministers of Education Organization [SEAMEO] 2020). These results are much lower than those of children in other countries in the region. Basic mathematics skills are necessary for children to solve problems in the context of their daily interests or important issues. Identifying the predictive role of kindergarteners’ EFs for later mathematics achievement within the Laotian capital could therefore supply essential baseline data relevant to future national policies and local educational efforts.

Empirical research on young children's EF development in Laos remains very limited. To our knowledge, prior psychological research on young Laotian children has not examined the links between socialization practices, children's EFs, and later mathematics skills. For example, Gomez et al. (2022) examined aspects of young Laotian children's development, including EFs, but did not address these specific associations. The present study extends the cultural and sociodemographic scope of research on early EF development by examining these associations in young Laotian children.

Accordingly, the present study has two aims. The first aim is to investigate how the associations between socialization practices (autonomy support, intrusive control) and early EFs in the Laotian capital compare to those widely reported in individualistic cultures within more‐EIRD contexts (Greenfield 2016). To contextualize these links, we first benchmark the frequency of these practices among Laotian capital caregivers against reference data from an individualistic culture within a more‐EIRD context (Belgium, Roskam et al. 2014) using the identical questionnaire. The second aim is to examine whether Laotian capital young children's EFs would predict their mathematics performance one year later.

Our hypotheses were as follows: First, regarding the associations between socialization practices and early EFs, following the mixed results found in collectivistic cultures, we hypothesized that autonomy support would show no or positive associations, and that intrusive control would show no or negative associations in the Laotian capital. To contextualize these links, we hypothesized that socialization practices in our Laotian sample would reflect traditional cultural values, characterized by less frequent autonomy support and more frequent intrusive control compared to the Belgian reference sample. Concerning the longitudinal association between early EFs and later mathematics achievement, we assumed that this positive association would also be observed in the Laotian capital. The first aim was examined in Study 1 at Time 1, and the second aim was investigated in Study 2 at Time 2.

## 2 Study 1

Study 1 examined the relationships between young children's EFs and socialization practices in the Laotian capital to see how they compare with those widely reported in individualistic cultures within more‐EIRD contexts (Greenfield 2016). As a preliminary step to contextualize these links, we benchmarked the frequency of these practices among Laotian caregivers against reference data from Belgium.

### 2.1 Method

#### 2.1.1 Participants

The study was conducted in one public and one private kindergarten in the Laotian capital. Children and their primary caregivers (co‐residing caregivers with the longest caregiving time) were recruited during meetings, drop‐offs, or pick‐ups. Caregivers provided written informed consent, and children gave oral assent. Of the 100 enrolled dyads, 20 were excluded before the initial sampling selection due to missing child tasks from absence (*n* = 17), missing caregiver surveys (*n* = 1), or age ineligibility (*n* = 2; Figure 1). From the initial sample (*N* = 80), 14 dyads were further excluded: complete task refusal (*n* = 1), performing only one EF task (*n* = 2; inclusion: ≥ 2 tasks), and caregiver‐reported developmental concerns (*n* = 11). To target typically developing children in a setting where formal medical diagnosis is limited, we asked caregivers to report developmental concerns based on a single questionnaire item (“Does your child exhibit any specific characteristics in their development?” [Yes/No]), supplemented with verbal examples (e.g., language, learning, and social delays). The final analytic sample comprised 66 dyads. While smaller than an a priori target (*N* = 84, calculated for *r* = 0.30 based on prior literature (Bernier et al. 2012; Matte‐Gagné and Bernier 2011; Matte‐Gagné et al. 2015), a sensitivity analysis using G*Power (v3.1.9.7) indicated that *N* = 66 (*α* = 0.05, power = 0.80; 4 total predictors, 1 tested for the main effects of socialization practices) provided sufficient sensitivity to detect a small‐to‐medium effect size (*f^2^* = 0.12 or larger; Cohen 2013).

**FIGURE 1:**
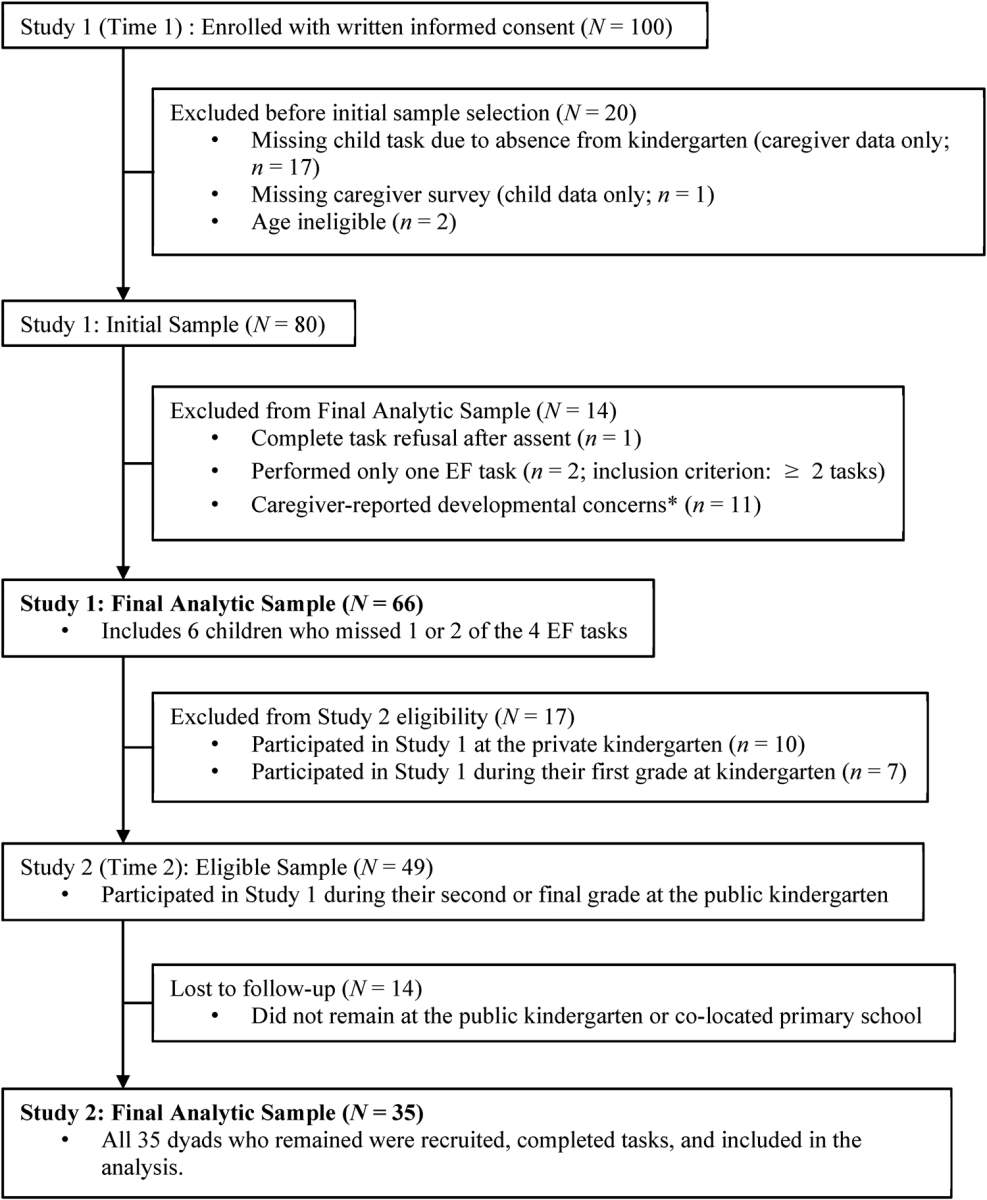
Participant Flow. EF = executive functions. *Based on a single caregiver questionnaire item regarding developmental characteristics/delays, supplemented with verbal examples.

The final 66 pairs (56 public, 10 private) included 27 boys and 39 girls (*M_age_* = 54.8 months, *SD* = 10.4, range = 37–78) and 66 primary caregivers (25 fathers, 35 mothers, one grandfather, four grandmothers, and one aunt; *M_age_* = 37.4 years, *SD* = 9.5, range = 22–65). Most caregivers were Laotian (*N* = 64), along with two Japanese caregivers fluent in Lao. The caregivers’ education levels were: no education (4.6%), nursery/kindergarten (4.6%), primary (9.1%), lower secondary (16.7%), upper secondary (22.7%), technical‐vocational diploma (21.2%), bachelor's (10.6%), master's (7.6%), and doctoral degree (3.0%), which was higher than the national average for ages 25–59 (Lao Statistics Bureau 2015). Household economic status, estimated using 13 consumer asset indicators (Lao Statistics Bureau 2015; UNICEF and Government of Laos 2020; Appendix 1), was 16.1% higher than the national average and 6.6% higher than the urban average (Lao Statistics Bureau 2015). A composite SES score, created by averaging the *z*‐scores of household economic status and caregiver education (which were correlated, *r* = 0.32, *p* = 0.009, 95% Confidence Interval (CI) [0.08, 0.52]), was used for the analyses. Children lived with an average of 3.56 adults (*SD* = 2.85, range = 1–15) and 1.27 siblings (*SD* = 1.00, range = 0–6) per household.

This study was approved by the Ethical Review Committee of the Kyoto University Psychological Science Unit (Approval#: 5‐P‐8).

#### 2.1.2 Tasks for Young Children

EF tasks were selected based on cultural and linguistic appropriateness, minimal time pressure or computerization, as most Laotian young children are not used to them, inclusion of both cool and hot EF components, and prior use in EIRD contexts:

1. **Hand Game**. (Hughes 1998) Children made a “rock” gesture to the experimenter's finger and vice versa across 15 pseudorandom trials. The correct responses were recorded and standardized.
2. **Black/White**. (Simpson and Riggs 2005) Children said “black” to a white card and “white” to a black card across 14 pseudorandom trials. The sum of the correct responses from the final practice (two trials) and test sessions was standardized.
3. **Dimensional Change Card Sorting**. (DCCS; Moriguchi and Shinohara 2018) Using target boxes (yellow house, green car) and eight test cards (four yellow cars, four green houses), children sorted cards by color, then shape, and finally alternated across three phases. The switching success rate was calculated and standardized.
4. **Gift Delay**. (Kochanska et al. 1996) Children were asked not to peek while the experimenter wrapped a gift for 60 seconds. Behavior was coded for strategy (2 = did not look, 1 = looked over shoulder, 0 = looked back) and latency to look. Interrater reliability (26% of the sample) was perfect. The strategy and latency scores were strongly correlated (*r* = 0.96, *p* < 0.001, 95% CI [0.93, 0.97]; *r*_s_ = 0.98, *p* < 0.001, 95% CI [0.95, 0.99]), which were standardized and averaged.
5. **Language Task**. (Appendix 2) A receptive vocabulary task adapted for Lao, based on the Picture Vocabulary Test–Revised (Ueno et al. 2008). Across 12 trials, children selected one picture matching the spoken Lao word among four pictures. The correct responses were summed.
  In the Hand Game, Black/White, DCCS, and language tasks, children who failed to pass the practice session did not proceed to the test. However, they received a score of zero (or one for the Black/White practice [pass ≥ 2 trials]) to differentiate them from non‐performing children.
6. **Procedure**. Task sessions (15–20 min) were administered individually in a quiet room by two trained Laotian experimenters using a standardized Lao script, supervised and videotaped by the first author. The fixed task order was as follows: (a) Hand Game (cool EFs), (b) Black/White (cool EFs), (c) DCCS (cool EFs), (d) Gift Delay (hot EFs), and (e) Language.

#### 2.1.3 Questionnaire for Caregivers

Primary caregivers completed the questionnaire on socialization practices (Appendix 3) at kindergarten or at home, with individual instructions provided by the experimenter. The questionnaire was a modified version of the *Évaluation des Pratiques Éducatives Parentales: Kindergarten and Primary School Form* (EPEP‐PPSF; Meunier and Roskam 2009). The questionnaire was translated into Lao, back‐translated, and randomized. To improve readability for caregivers with limited literacy, we reduced the scale from nine domains (40 items) to six domains (27 items) rated on a five‐point Likert scale (1 = never to 5 = always). Among these, Autonomy Support (perspective‐taking, respecting pace, encouraging participation), and Discipline, operationalized here as Intrusive Control (control involving intrusiveness, pressure, and domination), served as the primary, hypothesis‐driven focal domains, as they were expected to reflect core cultural differences in socialization practices between individualistic and collectivistic cultures. The remaining four domains were included for exploratory analyses.

#### 2.1.4 Analysis

Statistical analyses were performed in R (v4.4.2) using the tidyverse, psych, effectsize, and BayesFactor packages. Across Studies 1 and 2, all significance tests were two‐tailed. Multiplicity in frequentist multi‐test families was controlled using the Holm method, with only *p*‐values adjusted; 95% CIs were left unadjusted. Unadjusted statistics were reported for single‐pair correlations, preliminary checks, hierarchical regressions, effect sizes (*d*), and Bayesian estimation. To robustly evaluate null findings in socialization‐EF relationships (First aim), Bayesian multiple regression analyses were planned to complement non‐significant frequentist regressions.

### 2.2 Results

Preliminary analyses revealed no significant differences between the two kindergartens in children's composite EF scores (for calculation details of the EF score, see the subsequent section)—either uncontrolled (*p* = 0.066, Hedges’*g* = 0.64, 95% CI [−0.04, 1.29]) or controlling for covariates (age, age squared, and language: *p* = 0.766, *b* = −0.09, 95% CI [−0.69, 0.51]). Similarly, no significant differences between sites were found for socialization practices (autonomy support: *p* = 0.081, Hedges’ *g* = 0.52, 95% CI [−0.06, 1.08]; intrusive control: *p* = 0.769, Hedges’*g* = −0.10, 95% CI [−0.74, 0.55]; Table 1). For this and all subsequent analyses, socialization practices were analyzed simultaneously, and the results for the remaining four domains are provided in Appendices 4–9. Sites differed in children's language (*p* < 0.001, Hedges’*g* = 1.53, 95% CI [0.71, 2.33]) and SES (*p* < 0.001, Hedges’*g* = 1.44, 95% CI [0.62, 2.24]). A follow‐up regression showed that the site difference in language remained robust even when controlling for age and EFs (*p* < 0.001, *b* = −2.68, 95% CI [−4.17, −1.18]); thus, language was controlled in all subsequent EF‐related analyses. Conversely, there was no detectable association between SES and EFs, either at the zero‐order level (*p* = 0.956, *r* = −0.01, 95% CI [−0.25, 0.24] or when controlling for age and language (*p* = 0.233, *r* = −0.15, 95% CI [−0.39, 0.10]). No other demographic variables differed by site (*p*s > 0.050). Although site‐specific differences cannot be fully ruled out given the small and unequal sample sizes across sites (particularly *n* = 10 in the private kindergarten), data from both sites were pooled for primary analyses while statistically controlling for relevant covariates.

**TABLE 1:** Demographic, Socialization, and Task Homogeneity Verification between Kindergartens

	Private kindergarten (*N* = 10)		Public kindergarten (*N* = 56)		Analysis of homogeneity				
Variable	*N*	*M (SD)* or Count (%)	*N*	*M (SD)* or Count (%)	Mean difference	*t*	*df*	*p*	Hedges' *g* or Cramer's *V* [95% CI]
Age (months)	10	60.30 (13.77)	56	53.80 (9.55)	6.50	1.43	10.60	0.181	0.51 [−0.23, 1.23]
EF task performance (*z*)	10	0.55 (0.94)	56	−0.10 (0.99)	0.65	2.01	12.80	0.066	0.64 [−0.04, 1.29]
Language score	9	10.33 (1.80)	55	6.76 (2.53)	3.57	5.17	13.78	< 0.001	1.53 [0.71, 2.33]
Primary caregiver age	10	41.70 (10.32)	56	36.64 (9.28)	5.06	1.45	11.75	0.174	0.48 [−0.21, 1.15]
Socioeconomic status	10	0.90 (0.64)	56	−0.16 (0.73)	1.06	4.67	13.53	< 0.001	1.44 [0.62, 2.24]
Co‐residing older siblings	10	0.90 (1.29)	56	0.91 (0.82)	−0.01	−0.03	10.33	0.980	−0.01 [−0.72, 0.70]
Total co‐residing siblings	10	1.50 (1.78)	56	1.23 (0.81)	0.27	0.47	9.67	0.651	0.18 [−0.58, 0.93]
Total co‐residing adults	10	2.80 (0.92)	56	3.70 (3.05)	−0.90	−1.79	48.53	0.080	−0.39 [−0.83, 0.05]
Intrusive Control	10	2.56 (0.85)	56	2.65 (0.78)	−0.09	−0.30	11.92	0.769	−0.10 [−0.74, 0.55]
Autonomy Support	10	3.97 (0.75)	56	3.45 (1.13)	0.52	1.86	17.49	0.081	0.52 [−0.06, 1.08]
Sex (Male / Female)	10	5 (50.0%) / 5 (50.0%)	56	22 (39.3%) / 34 (60.7%)	—	—	—	0.729	0.00 [0.00, 1.00]*

*Note*: EF = executive functions. *t* = Welch's *t*‐statistic; *p*‐values and 95% CIs are unadjusted to preserve power for homogeneity detection. *Effect size for Sex is represented by Cramer's *V* [95% CI]. For socialization practices, all six domains were simultaneously analyzed to evaluate demographic homogeneity, with the two primary domains reported here and the remaining four domains provided in Appendix 4.

#### 2.2.1 Children's Performance in EF and Language Tasks

Table 2 presents descriptive statistics. As the data did not meet the assumption of normality, non‐parametric Spearman's rank correlations were calculated among the EF tasks. Results revealed a consistent pattern of positive but generally modest intercorrelations, with some coefficients reaching statistical significance (*r*_s_s ranged from 0.19 to 0.39, *p*_adj_s ranged from 0.011 to 0.181; Table 3). The EF battery demonstrated modest internal consistency (Cronbach's *α* = 0.63, standardized *α* = 0.65, 95% CI [0.48, 0.77]), which was not improved by deleting any task(remaining standardizedαranged from 0.54 to 0.64). A principal component analysis supported a single‐component structure (eigenvalue = 1.89) accounting for 47.2% of the total variance, with all four tasks loading strongly (factor loadings ranged from 0.61 to 0.78).

**TABLE 2:** Means, Standard Deviations, and Ranges in the Scores of EF and Language Tasks

Descriptive statistics	Hand Game	Black/ White	DCCS (Switch success rate)	Gift Delay (Strategy)	Gift Delay (Latency (seconds))	Language
*N*	62	61	66	65	65	64
*M*	7.68	8.30	0.55	1.34	44.35	7.27
*SD*	5.61	6.42	0.19	0.94	23.23	2.73
Range	0–15	0–16	0–1	0,1,2	0–60	1–12

*Note*. EF = executive functions. DCCS = Dimensional Change Card Sorting task.

**TABLE 3:** Spearman's Rank Correlation Coefficients Examining the Relations between the Four EF Tasks

Task	1	2	3	4
1. Hand Game (*N* = 62)				
2. Black/White (*N* = 61)	0.22 (*N* = 61) *p*_adj_ = 0.181 [−0.04, 0.45]			
3. DCCS (*N* = 66)	0.19 (*N* = 62) *p*_adj_ = 0.181 [−0.07, 0.42]	0.36 (*N* = 61) *p*_adj_ = 0.025 [0.11, 0.56]		
4. Gift Delay (*N* = 65)	0.27 (*N* = 61) *p*_adj_ = 0.115 [0.01, 0.49]	0.39 (*N* = 60) *p*_adj_ = 0.011 [0.15, 0.59]	0.30 (*N* = 65) *p*_adj_ = 0.060 [0.05, 0.51]	

*Note*: EF = executive functions. DCCS = Dimensional Change Card Sorting task.

*p*‐values were adjusted for multiple comparisons across the family of six pairwise tests using the Holm method. 95% CIs were unadjusted.

Consequently, a composite EF score was created by averaging the *z*‐transformed scores across the four tasks, which were standardized for subsequent analyses. For six children who missed one or two EF tasks, composite scores were calculated using available tasks; four missed both Hand Game and Black/White, and two missed either Black/White or Gift Delay (the latter two also missed the language task). As five of these six children were under age four, their performance may have been affected by initial nervousness or fatigue toward the end of the session.

EF composite scores correlated positively with age in months (*r* = 0.58, *p*_adj_ < 0.001, 95% CI [0.40, 0.72]; Figure 2A). Testing for non‐linear developmental trends, a quadratic model explained significantly more variance than a linear model (Δ*R^2^* = 0.05, *F* (1,63) = 5.63, *p* = 0.021), showing a significant negative quadratic effect of age (*b* = −0.00, *p* = 0.021, adjusted *R^2^* = 0.37). EF performance also correlated with language ability (*r* = 0.59, *p*_adj_ < 0.001, 95% CI [0.41, 0.73]; Figure 2B), and age correlated with language (*r* = 0.42, *p*_adj_ <.001; 95% CI [0.20, 0.61]). Holm adjustment was applied within these variables. Sociodemographic variables (co‐residing older siblings, total siblings, and adults) were also uncorrelated with EFs (*r*s ranged from −0.02 to 0.16, *p*_adj_s ≥ .611, 95% CI [−0.26, 0.39]). Holm adjustment was applied within these sociodemographic variables.

**FIGURE 2:**
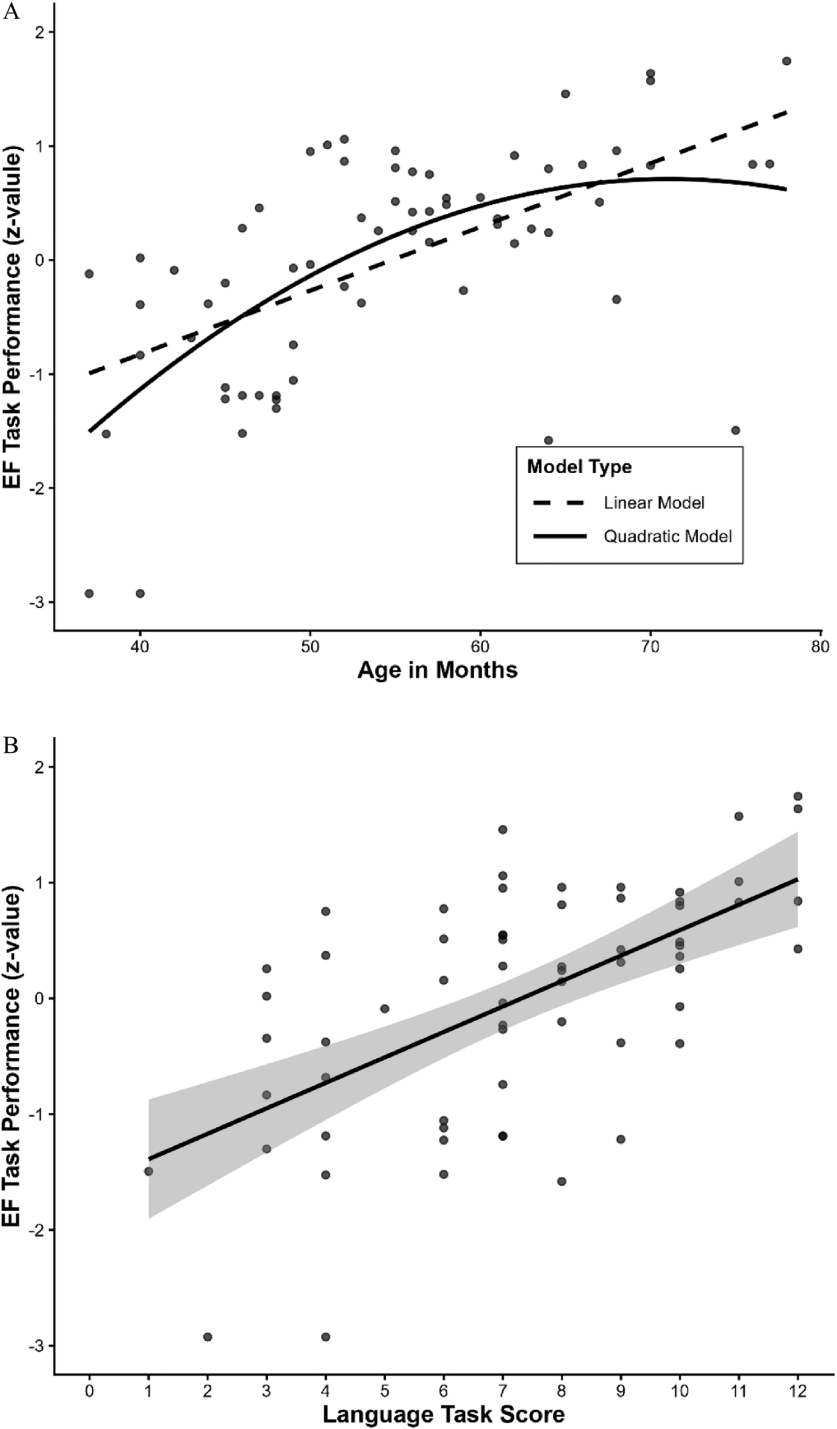
(A) Relationship between Children's Age and Executive Function (EF) Task Performance. The EF Task Performance score represents the mean z‐score calculated across the four EF tasks (standardized again as a composite z‐score). (B) Relationship between children's language and executive function (EF) task performances.

#### 2.2.2 Socialization Practices: Laos vs. Belgium

To descriptively contextualize Laotian socialization practices, we compared them with French‐speaking Belgian reference data (Roskam et al. 2014; Belgium individualism index = 81, Culture Factor Group 2023) using the same questionnaire. Belgian inhibition data were excluded due to measurement and composite differences, focusing our comparison solely on socialization practices. To minimize relational and age‐related confounding, we utilized longitudinal data from Belgian mothers, who are primary caregivers, at Time 1 (*N* = 348). Their children's age (*M_ag_*_e_ = 55.3 months, *SD* = 11.14, range = 31–91) closely matched our Laotian sample (*M_age_* = 54.8 months, *SD* = 10.4, range = 37–78). The Belgian sample comprised families recruited from nursery schools (70%) or a university hospital in the capital where children were screened out for developmental or neurological issues (30%).

As shown in Table 4 with descriptive statistics, Laotian caregivers reported levels of autonomy support comparable to those of Belgian caregivers (*d* = −0.05, 95% CI [−0.31, 0.21]). In contrast, intrusive control was reported less frequently by Laotian caregivers than by Belgian caregivers (*d* = −1.12, 95% CI [−1.39, −0.84]. Preliminary analyses revealed no associations between these socialization domains and children's age or language, either before or after controlling for the other variable (all *p*_adj_s > 0.506; Appendices 5–6).

**TABLE 4:** Means and Standard Deviations of Socialization Practices in Laos and Belgium, and the Effect Sizes

Context	Descriptive statistics	Intrusive Control	Autonomy Support
Laos	*M*	2.63	3.52
(*N* =66)	*SD*	0.79	1.10
Belgium	*M*	3.43	3.56
(*N* = 348)	*SD*	0.70	0.71
Effect sizes	*d* 95% CI	−1.12 [−1.39, −0.84]	−0.05 [−0.31, 0.21]

*Note*: For Belgium, the maternal data from Roskam et al. (2014, p. 173, Table 1, T1) were cited. Responses are on a five‐point Likert scale from never (1) to always (5). To ensure comprehensive data transparency, this table displays the two primary domains of interest, while the descriptive statistics and effect sizes for the remaining four socialization domains are provided in Appendix 7. 95% CIs were unadjusted.

#### 2.2.3 Associations between Children's EF Performance and Socialization Practices

Zero‐order Pearson correlations showed that EFs were not significantly correlated with autonomy support (*r* = −0.02, *p*_adj_ = 0.999, 95% CI [−0.26, 0.22]) or intrusive control (*r* = 0.06, *p*_adj_ = 0.999, 95% CI [−0.18, 0.30]; Table 5). Next, separate multiple regressions were conducted to predict EFs while controlling for child age, age squared, and language. Although both models explained substantial variance (autonomy support: *R* ^2^ = 0.58, *F* (4, 59) = 20.05, *p* < 0.001; intrusive control: *R* ^2^ = 0.55, *F* (4, 59) = 17.70, *p* < 0.001), there was no significant main effect for either autonomy support (*b* = −0.17, *p*_adj_ = 0.162, 95% CI [−0.32, −0.01]) or intrusive control (*b* = −0.04, *p*_adj_ = 0.999, 95% CI [−0.26, 0.19]). To complement these frequentist null findings—which merely indicate a failure to reject the null hypothesis—we conducted Bayesian multiple regressions controlling for the same covariates. For autonomy support, the Bayesian approach showed anecdotal evidence for a weak negative association (*BF*_10_ = 1.54, posterior mean = −0.16, *SD* = 0.08, 95% credible interval [−0.31, −0.00]). In contrast, for intrusive control, the data actively supported a null association (*BF*_10_ = 0.23, posterior mean = −0.04, *SD* = 0.11, 95% credible interval [−0.25, 0.18]), representing moderate evidence that the data are approximately four times more compatible with a null model than with the specific alternative model.

**TABLE 5:** Zero‐order Correlation Analysis between EF Task Performance and Socialization Practices, as well as Regular and Bayesian Multiple Regression Analyses Predicting EF Task Performance

Analysis	Statistical summary	Intrusive Control	Autonomy Support
Zero‐order (*N* = 66)	*r p*_adj_ 95% CI	0.06 0.999 [−0.18, 0.30]	−0.02 0.999 [−0.26, 0.22]
Multiple regression (*N* = 64)	*b* (*SE*) *p*_adj_ 95% CI *F R* ^2^ (adjusted *R* ^2^)	−0.04 (0.11) 0.999 [−0.26, 0.19] 17.70 0.55 (0.51)	−0.17 (0.08) 0.162 [−0.32, −0.01] 20.05 0.58 (0.55)
Bayesian multiple regression (*N* = 64)	Posterior Mean (*SD*) *BF*_10_ 95% Credible Interval	−0.04 (0.11) 0.23 [−0.25, 0.18]	−0.16 (0.08) 1.54 [−0.31, −0.00]

*Note*: EF = executive functions. In both regular and Bayesian multiple regressions, children's age, age squared, and language ability were controlled for. The *p‐*values of frequentist analyses (zero‐order correlations and regression coefficients) were adjusted using the Holm method across all six measured socialization domains (including the four domains reported in Appendix 8) to maintain statistical rigor. 95% CIs were unadjusted. Bayesian statistics reflect unadjusted posterior distributions.

### 2.3 Discussion

The first aim of this study was to examine how the associations between socialization practices (autonomy support and intrusive control) and young children's EFs in the Laotian capital—a rapidly transitioning sociodemographic context within a traditionally collectivistic and less‐EIRD (Greenfield 2018) context—compare with those widely reported in individualistic cultures within more‐EIRD contexts (Greenfield 2016). To contextualize these socialization‐EF links, we first benchmarked the self‐reported frequency of caregiver practices in the Laotian sample against reference data from Belgium (Roskam et al. 2014) using the identical questionnaire. Regarding autonomy support, we initially hypothesized that the Laotian sample would practice it less frequently than the Belgian reference sample. Contrary to this expectation, autonomy support was reported at a comparable level in the two contexts. Although speculative and grounded primarily in theoretical expectations (Greenfield 2016), this finding may reflect shifting cultural values of Laotian capital caregivers along the collectivism‐individualism spectrum, which can be theorized to relate to sociodemographic transitions. However, autonomy support showed an anecdotal negative trend rather than a hypothesized positive or null association. Given the highly marginal nature of this evidence, this unpredicted trend must be interpreted with extreme caution. While purely tentative, one speculative possibility is that autonomy support, as measured here, might not yet be fully embedded in the broader cultural norms and meanings of the sociodemographically changing Laotian capital. Previous research suggests that cultural norms and meanings may moderate associations between socialization practices and child development because caregivers and children interpret caregiving not only in their own relationships but also in the broader socialization ecology (Cho et al. 2023; Lansford 2022). From this perspective, it is possible that autonomy support might not necessarily function as a salient or consistent signal for children's self‐regulatory development in this transitional context, even when reported by caregivers.

Regarding intrusive control, diverging from our hypothesis, it was practiced less frequently in the Laotian sample than in the Belgian reference sample. As for its relationship with EFs, consistent with our hypothesis, the data provided moderate evidence supporting a null association with young children's EFs. Although not directly measured in the present study, one speculative possibility for both the lower frequency and the lack of a negative association with EFs may relate to the structural nature of socialization in Laos. Here, socialization responsibilities are often distributed across extended family members and community networks, which could conceivably buffer caregivers’ emotional strain and reliance on reactive, stress‐induced control strategies (Roskam et al. 2024). Under such circumstances, intrusive control may be employed more selectively and intentionally, though this hypothesis remains empirically untested. Moreover, as suggested by Cho et al. (2023), the psychological meanings and functions of socialization practices may vary across cultural frameworks. In collectivistic cultures, socialization practices that appear controlling or intrusive from an individualistic standpoint might instead be perceived by children as legitimate guidance or genuine expressions of care and involvement (Chao 1994; Pomerantz and Wang 2009). Consequently, intrusive control may not undermine children's EF development in the same way as reported in individualistic cultures within more‐EIRD contexts, consistent with the possibility that socialization‐EF relations vary across sociocultural contexts.

## 3 Study 2

Study 2 investigated whether young children's EFs in the Laotian capital predict their mathematics performance one year later.

### 3.1 Method

#### 3.1.1 Participants

From the Study 1 analytic sample (*N* = 66), 49 children who participated during their second or final kindergarten grade at the public kindergarten were eligible for Study 2 (Figure 1). Of these, 35 children remained in the kindergarten (final grade) or entered the co‐located primary school (first grade). All of them were recruited during drop‐offs and pick‐ups and completed Study 2 (13 boys and 22 girls; *M_age_* = 68.2 months, *SD* = 8.8, range = 54–90). Primary caregivers provided written informed consent, while children provided oral assent. The sample included 17 kindergarteners (10 boys, 7 girls; *M_age_* = 61.5 months, *SD* = 5.0, range = 54–75) and 18 primary school students (3 boys, 15 girls; *M_age_* = 74.6 months, *SD* = 6.5, range = 66–90). To maintain consistency and avoid confounding attrition bias with shifting family dynamics, household characteristics were evaluated based on Time 1 baseline profiles. At baseline, primary caregivers (all Laotian; *M_age_* = 37.1 years, *SD* = 9.3, range = 22–61) included 13 fathers, 19 mothers, one grandfather, and two grandmothers. Caregiver education levels were: no education (8.6%), nursery/kindergarten (2.9%), primary (11.4%), lower secondary (25.7%), upper secondary (20.0%), technical‐vocational diploma (14.3%), bachelor's (11.4%), and doctoral degree (5.7%).

A post‐hoc sensitivity analysis (G*Power v3.1.9.7) indicated that with *N* = 35, α = 0.05, and power = 0.80 (4 total predictors, 1 tested for the main effect of EFs), the study was sensitive to a medium effect size of *f^2^* = 0.24 or larger (Cohen 2013). To evaluate attrition bias, baseline characteristics were compared between Study 2 participants (*N* = 35) and non‐participants (*N* = 14) among the targeted children (*N* = 49; Table 6). Although a significant difference between the groups was found only in the number of co‐residing adults (*p* = 0.008, Hedges’ *g* = 0.71, 95% CI [0.19, 1.24]), the possibility of a systematic attrition bias cannot be completely excluded. To address this, the number of co‐residing adults (*M* = 4.40, *SD* = 3.57, range = 2–15) was included as a covariate in subsequent regression models.

**TABLE 6:** Comprehensive Attrition Analysis for the Time‐2 Eligible Sample: Baseline Demographic, Socialization, and Task Comparison between Time‐1‐only and Time‐2 Participants

	Time‐2 participants		Time‐1‐only participants		Attrition analysis				
Variable	*N*	*M* (*SD*) or Count (%)	*N*	*M* (*SD*) or Count (%)	Mean difference	*t*	*df*	*p*	Hedges' *g* or Cramer's *V* [95% CI]
Age (months)	35	56.71 (8.47)	14	53.79 (7.73)	2.93	1.17	26.18	0.254	0.35 [−0.25, 0.94]
EF task performance (*z*)	35	0.08 (1.03)	14	−0.20 (0.92)	0.28	0.92	26.64	0.368	0.27 [−0.32, 0.86]
Language score	35	6.94 (2.39)	14	7.79 (2.19)	−0.84	−1.19	26.05	0.247	−0.36 [−0.95, 0.24]
Primary caregiver age	35	37.14 (9.30)	14	35.07 (10.21)	2.07	0.66	22.13	0.517	0.20 [−0.41, 0.82]
Socioeconomic status	35	0.02 (0.75)	14	−0.04 (0.85)	0.06	0.24	21.46	0.811	0.08 [−0.54, 0.69]
Co‐residing older siblings	35	1.00 (0.91)	14	0.71 (0.61)	0.29	1.27	35.48	0.211	0.36 [−0.20, 0.92]
Total co‐residing siblings	35	1.34 (0.87)	14	1.07 (0.73)	0.27	1.11	28.54	0.276	0.33 [−0.26, 0.91]
Total co‐residing adults	35	4.40 (3.57)	14	2.43 (1.40)	1.97	2.77	47.00	0.008	0.71 [0.19, 1.24]
Intrusive Control	35	2.63 (0.78)	14	2.76 (0.83)	−0.13	−0.50	22.89	0.623	−0.15 [−0.76, 0.46]
Autonomy Support	35	3.50 (1.10)	14	3.69 (1.24)	−0.19	−0.49	21.62	0.631	−0.15 [−0.77, 0.46]
Sex (Male/Female)	35	13 (37.1%) / 22 (62.9%)	14	6 (42.9%) / 8 (57.1%)				0.754	0.00 [0.00, 1.00]*

*Note*: EF = executive functions. *t* = Welch's *t*‐statistic. *p*‐values and 95% CIs were unadjusted to preserve power for attrition bias detection. Although all six socialization domains were analyzed together to maintain consistency, the data for the remaining four domains are presented in Appendix 9. *Effect size for Sex is represented by Cramer's *V* [95% CI].

This study was approved by the Ethical Review Committee of the Kyoto University Psychological Science Unit (Approval#: 6‐P‐15).

#### 3.1.2 Mathematics Task

Children's mathematics ability was assessed at the beginning of the academic year. A 10‐item test (Appendix 10) was developed based on official Laotian mathematics textbooks for kindergarten final graders (Research Institute for Educational Science, Ministry of Education and Sports 2016), with item design informed by the numerical reasoning subtests of Japanese K‐ABC II (Japanese version of K‐ABC Production Committee, 2013). The test was designed to be relatively short to fit Laotian young children who are generally unfamiliar with formal testing situations. The test assessed mathematics skills, including counting, cardinality, numeral recognition, magnitude comparison, spatial relations, and simple addition and subtraction. Children responded orally, showing with fingers, or by pointing—with the number of correct answers (0–10) serving as a composite performance score.

The 10‐item task demonstrated good internal consistency (Cronbach's *α* = 0.82, 95% CI [0.74, 0.90]; mean inter‐item *r* = 0.31), with item deletion yielding similar reliability (*α*s = 0.78–.83). Although the task covers multiple mathematical subdomains, each is represented by only one or two items. Due to this brief item composition and sample size constraints, robustly evaluating a multidimensional structure or separating subscales was not methodologically feasible. Therefore, the sum score was used as an aggregate index of overall early mathematics performance, rather than a strictly validated unidimensional scale.

#### 3.1.3 Procedures

The mathematics task was individually administered in a quiet room at each participant's institution. The same trained Laotian experimenter who conducted the tasks in Study 1 administered the test using a standardized script. All sessions were supervised and video‐recorded by the first author.

### 3.2 Results

Statistical analyses were performed using R (v4.4.2) with the tidyverse, psych, and effectsize packages.

For mathematics performance (*N* = 35), the mean score was 5.51 (*SD* = 2.64, range = 0–10; Table 7). Mathematics performance was positively correlated with age (*r* = 0.54, *p*_adj_ < 0.001, Holm‐adjusted for children's age and EFs; 95% CI [0.26, 0.74]), and Time 1 composite EF scores (*r* = 0.57, *p*_adj_ < 0.001, 95% CI [0.30, 0.76]), but not with SES (*r* = −0.18, *p*_adj_ = 0.288, Holm‐adjusted for SES and co‐residing adults; 95% CI [−0.49, 0.16]) or co‐residing adults (*r* = 0.26, *p*_adj_ = 0.259, 95% CI [−0.08, 0.55]). A hierarchical multiple regression was conducted to examine the unique contribution of EFs, alongside the incremental roles of age, co‐residing adults, and SES (Table 8). Model 1, including EFs only, explained a significant 32.9% of the variance in mathematics performance, *F* (1, 33) = 16.16, *p* < 0.001, with EFs significantly predicting mathematics performance (*β* = 0.57, *p* < 0.001). Adding age in Model 2 increased the explained variance to 41.6%, *F* (2, 32) = 11.40, *p* < 0.001, and both EFs (*β* = 0.40, *p* = 0.015) and age (*β* = 0.34, *p* = 0.036) were significant positive predictors. Neither the addition of co‐residing adults in Model 3 (Δ*R^2^* = 0.00, Δ*F* (1, 31) = 0.00, *p = 0*.969) nor SES in Model 4 (Δ*R^2^* = 0.00, Δ*F* (1, 30) = 0.23, *p = 0*.638) significantly improved model fit. In the final model, EFs remained the only significant predictor (*β* = 0.40, *p* = 0.018), whereas age (*β* = 0.32, *p* = 0.089), co‐residing adults (*β* = 0.02, *p* = 0.909), and SES (*β* = −0.07, *p* = 0.638) were non‐significant.

**TABLE 7:** Means and Standard Deviations of the Mathematics Task Scores

Question	*N*	*M*	*SD*	*Min*	*Max*
Q1	35	0.89	0.32	0.00	1.00
Q2	35	0.60	0.50	0.00	1.00
Q3	35	0.43	0.50	0.00	1.00
Q4	35	0.77	0.43	0.00	1.00
Q5	35	0.29	0.46	0.00	1.00
Q6	35	0.20	0.41	0.00	1.00
Q7	35	0.91	0.28	0.00	1.00
Q8	35	0.83	0.38	0.00	1.00
Q9	35	0.31	0.47	0.00	1.00
Q10	35	0.29	0.46	0.00	1.00
Math sum	35	5.51	2.64	0.00	10.00

**TABLE 8:** Hierarchical Multiple Regression Analyses Predicting Mathematics Performance.

Variable	*b (SE)*	*β*	*t*	*p*	95% CI
Model 1					
EF task performance (*z*)	1.51 (0.38)	.57	4.02	< .001	[0.75, 2.28]
Model summary	*R* ^2^ = .329, adj. *R* ^2^ = .308, *F* (1, 33) = 16.16, *p* < .001				
**Model 2**					
EF task performance (*z*)	1.06 (0.41)	.40	2.58	.015	[0.22, 1.90]
Age (months)	0.10 (0.05)	.34	2.19	.036	[0.01, 0.20]
Model summary	*R* ^2^ = .416, adj. *R* ^2^ = .380, *F* (2, 32) = 11.40, *p* < .001, Δ*R* ^2^ = .087, Δ *F* (1, 32) = 4.78, *p *= .036				
**Model 3**					
EF task performance (*z*)	1.06 (0.42)	.40	2.53	.017	[0.21, 1.92]
Age (months)	0.10 (0.05)	.34	1.98	.057	[‐0.00, 0.21]
Co‐residing adults	0.00 (0.11)	.01	0.04	.969	[‐0.23, 0.24]
Model summary	*R* ^2^ = .416, adj. *R* ^2^ = .360, *F* (3, 31) = 7.36, *p* < .001, Δ*R* ^2^ = .000, Δ*F* (1, 31) = 0.00, *p* = .969				
**Model 4**					
EF task performance (*z*)	1.07 (0.42)	.40	2.51	.018	[0.20, 1.93]
Age (months)	0.10 (0.05)	.32	1.76	.089	[‐0.02, 0.21]
Co‐residing adults	0.01 (0.12)	.02	0.12	.909	[‐0.22, 0.25]
Socioeconomic status	−0.25 (0.52)	‐.07	−0.48	.638	[‐1.32, 0.82]
Model summary	*R* ^2^ = .420, adj. *R* ^2^ = .343, *F* (4, 30) = 5.44, *p* = .002, Δ*R* ^2^ = .004, Δ*F* (1, 30) = 0.23, *p* = .638				

*Note: N* = 35. EF = executive functions. *p*‐values and 95% CIs for regression coefficients were unadjusted, as is standard for hierarchical regression models. 95% CIs are reported for unstandardized regression coefficients (*b*).

### 3.3 Discussion

Study 2 examined whether young children's EFs in the Laotian capital predict their mathematics performance one year later. Consistent with our hypothesis, Time 1 EFs positively predicted Time 2 mathematics performance. By empirically testing the boundary of this association within an understudied, transitional context, our findings contribute to extending the applicability of developmental theories. Moreover, in the Laotian capital, where mathematics achievements pose a significant national challenge (UNICEF and SEAMEO 2020), early EFs may play a foundational role in supporting mathematical learning. Children with higher levels of EFs are likely better equipped to maintain task goals, inhibit irrelevant responses, and flexibly shift between problem‐solving strategies (e.g., Emslander and Scherer 2022). Ultimately, these findings provide initial evidence relevant to future national policies and local efforts aimed at improving early mathematics acquisition.

## 4 General Discussion

Across two studies, the present study examined the associations of young children's EFs with socialization practices and later mathematics skills in the Laotian capital, a rapidly transitioning sociodemographic locale within a traditionally collectivistic and less‐EIRD (Greenfield 2018) setting. Together, the findings reveal a distinctive pattern: While the longitudinal association between EFs and early mathematics appears to extend to this context, the relations between socialization practices and EFs are consistent with the possibility that they may vary across sociocultural contexts.

First, regarding socialization practices, we observed a pattern that differs from typical assumptions derived largely from research conducted in individualistic cultures within more‐EIRD contexts (Greenfield 2016). Caregivers in the Laotian capital reported levels of autonomy support comparable to those reported by the Belgian reference sample. However, we found no clear evidence for a positive association between autonomy support and children's EFs, and evidence for a negative association was only anecdotal. While pending direct empirical validation, this unexpected comparability in frequency might reflect shifting cultural values among urban Laotian caregivers along the collectivism‐individualism continuum, theoretically aligned with sociodemographic shifts (Greenfield 2016). Regarding the lack of clear evidence for a positive association with EFs, one potential, highly tentative explanation is that autonomy support might not yet be fully embedded in widely shared cultural norms and meanings in the socialization ecology of the Laotian capital. Consequently, such practices might not necessarily function as a salient or consistent signal for children's EF development (Cho et al. 2023; Lansford 2022).

In addition, Laotian capital caregivers practiced intrusive control less frequently than their Belgian reference sample, and intrusive control was not associated with EFs. This finding is consistent with results from some other collectivistic contexts (Doan and Song 2022; Sun et al., 2025; Xie and Li 2022) and suggests the importance of considering socialization structures beyond parent‐child dyads. In Laos, socialization responsibilities are often shared within extended families and communities. While not directly measured in the present study, one speculative possibility is that this distributed caregiving network might help buffer caregivers’ stress, promoting more intentional and selective use of intrusive control rather than a chronic interactional style (Roskam et al. 2024). Within such a context, and as highlighted by Cho et al. (2023), the psychological meanings and functions of similar socialization practices may operate uniquely across different sociocultural contexts: practices that might be perceived as coercive in individualistic cultures may instead be interpreted by children in collectivistic cultures as legitimate guidance or expressions of care (Chao 1994; Pomerantz and Wang 2009).

Second, we found that early EFs predicted Laotian children's mathematics performance one year later. This result extends the previous findings—mostly conducted in more‐EIRD contexts—to the Laotian capital, providing additional evidence consistent with the possibility that the association between early EFs and later mathematics performance extends across diverse sociodemographic contexts.

Taken together, the present findings point to an important distinction between the socialization‐EF relations and the longitudinal association between EFs and later mathematics achievement. While how socialization practices relate to EFs may reflect sociocultural context, the role of EFs in supporting later mathematics skills provides additional evidence aligning with patterns established in previous research, pending further validation across diverse settings. These results underscore the importance of considering sociocultural contexts when modeling early socialization and EF development, while preserving a focus on relatively shared developmental mechanisms underlying early mathematical learning.

From an applied perspective, although interpreted with caution due to the limited sample size, this study provides preliminary evidence that supporting EFs in early childhood has the potential to yield long‐term benefits for mathematics achievement. At the same time, future efforts to support children's EFs might benefit from taking local socialization practices and social structures into account, rather than assuming that the socialization models derived from individualistic cultures within more‐EIRD contexts are directly transferable.

The limitations of this study include the following. First, the modest sample sizes from only two kindergartens (one private, one public) limit generalizability to the wider population in the Laotian capital, where kindergarten attendance is 63.8% (Lao Statistics Bureau 2018). However, post‐hoc sensitivity analyses (power = 0.80) indicated that Study 1 (*N* = 66) could detect effect sizes of *f* ^2　^ = 0.12 or larger, whereas Study 2 (*N* = 35) could detect effect sizes of *f* ^2^ = 0.24 or larger. Although relevant covariates that differed across sites (i.e., language ability) were statistically controlled for in primary analyses, potential site‐specific institutional practices—as well as the substantial sample size imbalance between the two sites—and non‐attendance effects remain unexamined. Future research should employ larger, randomized cluster samples including non‐attending children. Second, we interpreted our findings through Greenfield's theoretical framework, connecting sociodemographic EIRD contexts with cultural orientations, even though cultural orientations were not directly measured. Future research should incorporate validated cultural scales to empirically test these theoretical interpretations. Third, a small subset of children (*n* = 6) participated in only two or three of the four EF tasks mainly due to initial anxiety or fatigue. Although preliminary analyses supported modest internal consistency and a single‐factor structure, constructing composite scores from incomplete task sets remains a methodological constraint inherent to testing young children. Fourth, our mathematics task was intentionally brief (10 items) to minimize child burden. While internal consistency was high (α = 0.82), the small number of items per subdomain prevented us from evaluating a multidimensional structure. Future studies should explore refined item designs of item‐response approaches that can better capture subdomains within a brief format. Fifth, relying on self‐reported socialization measures rather than direct observations may have limited sensitivity to everyday interactional processes. Sixth, developmental exclusions relied on a single caregiver‐reported item rather than formal medical diagnoses, which represents a methodological limitation of our selection procedure in this context. Finally, we surveyed only primary caregivers, despite socialization in Laos typically involving multiple individuals. Future studies should account for extended family and community members.

## 5 Conclusions

This study provides novel empirical insights into the association between socialization practices and early EFs and the role of EFs in later mathematics skills in the Laotian capital, a rapidly shifting sociodemographic pocket in a traditionally collectivistic and less‐EIRD context. While extending previous evidence on the role of early EFs in subsequent mathematics achievement—thereby offering preliminary evidence to inform local educational strategies—our findings regarding socialization‐EF relations were consistent with the possibility that these links vary across sociocultural contexts. Specifically, we found only anecdotal evidence for a negative association between autonomy support and EFs, and moderate evidence for a null association between intrusive control and EFs. Tentatively, these findings suggest that future efforts by caregivers and teachers to support children's EFs may benefit from considering local cultural values and norms. Overall, the present study lays an empirical and theoretical foundation for future research and provides evidence relevant to future policy development aimed at fostering children's cognitive and academic development across diverse sociocultural contexts.

## Author Contributions

**Yoko Kajiyama**: conceptualization, methodology, software, data curation, investigation, formal analysis, visualization, resources, writing – original draft, writing – review and editing, project administration. **Yusuke Takahashi**: conceptualization, supervision, writing – review and editing. **Yusuke Moriguchi**: conceptualization, supervision, writing – review and editing, funding acquisition.

## Funding information

This research was supported by Japan Society for the Promotion of Science (KAKENHI) Grant Number B24K00486.

## Conflicts of Interest

The authors do not have any interests that might be interpreted as influencing the research.

## Supporting information




**Supporting information**: desc70302‐sup‐0001‐SuppMat.pdf

## Data Availability

The data that support the findings of this study are available on OSF via the following anonymized view‐only link for peer review: https://osf.io/z3xba/overview?view_only=0d2866b7539041d2b68b2e919cbf2c36
