# Supplementary material for: Executive Functions Among Kindergarteners in the Capital of Laos: Relations with Socialization Practices and Later Mathematics Skills
Source: Dev Sci. 2026 Nov 3;30(1):e70302. doi: 10.1111/desc.70302 (PMC13728889; doi:10.1111/desc.70302)
Supplement: Supplementary file 1 [file DESC-30-e70302-s001.pdf]

Appendix 1

Household Economic Status and Caregiver Education Household Economic Status

Household economic status was estimated using 13 consumer asset indicators as follows (Lao Statistics Bureau, 2015; UNICEF & Government of Laos, 2020): A tractor, a car, a motorbike, a television, a mobile phone, a PC/tablet, a washing machine, an air conditioner, an electric fan, a refrigerator, agricultural land, livestock, and an internet connection. One point corresponds to the possession of each of the 13 items. Therefore, the maximum score was 13.

Caregiver Education

Education Levels from which Caregivers were Asked to Select

Caregiver's educational attainment was divided into the following 15 categories to make it easy for participants to respond (World Bank, 2016): 1. No education, 2. Attendance at nursery, 3. Attendance at kindergarten, 4. Attendance at or completion of primary school, 5. Attendance at lower secondary school, 6. Completion of lower secondary school, 7. Attendance at upper secondary school, 8. Completion of upper secondary school, 9. Certificate in technical-vocational education and training (TVET), 10. Diploma in TVET, 11. Advanced Diploma in TVET, 12. Pre-bachelor's degree, 13. Bachelor's degree, 14. Master's degree, and 15. Doctoral degree.

Education Levels Used for the Analyses

In the analysis, the 15 levels were condensed to nine levels from 0 to 8 by combining items that are continuous or overlapped in terms of their corresponding age ranges, ensuring that all the groups were systematically ordered. Specifically, Level 0 corresponds to the original Level 1 (no education), Level 1 corresponds to the original Levels 2 and 3 (attended nursery or kindergarten), Level 2 corresponds to the original Level 4 (attended or graduated

from primary school), Level 3 corresponds to the original Levels 5 and 6 (attended or graduated from lower secondary school), Level 4 corresponds to the original Levels 7, 8, and 9 (attended or graduated from upper secondary school (including certificate in TVET), Level 5 corresponds to the original Levels 10, 11, and 12 (diploma or advanced diploma in TVET, or pre-bachelor's degree), Level 6 corresponds to the original Level 13 (Bachelor's degree), Level 7 corresponds to the original Level 14 (Master's degree), and Level 8 corresponds to the original Level 15 (Doctoral degree).

Appendix 2

Details of the Language Task

The authors designed this receptive vocabulary task to suit Laotian culture with the help of Laotian experimenters. Inspiration was drawn from an established test, specifically the Picture Vocabulary Test-Revised (Ueno et al., 2008). The test consists of 12 pages (24cm × 33cm), each of which features four colored pictures: the one that best matches the stimulus word and three distractors (see the table below). The stimulus words and the subject matter of the pictures were chosen to be familiar to Laotian children, being drawn from typical words used in the Laotian alphabet table, as well as from the contents of Laotian picture books and early-grade textbooks. The distractors were combined with the correct pictures to be appealing choices for children who were unsure of the correct answers. Moreover, the stimulus words were selected to enable the task to reflect differences in scores for children aged three to six by testing not only the name of an item (Practice & Q1) but also various linguistic concepts, including a specific part of an item (Q6), its category (Q2 and Q3), characteristics (Q4, Q5, and Q10), functions (Q7), purposes (Q8, Q9, Q11, and Q12).

Contents of the Task

**Question**	**Stimulus word**	**Picture 1 (upper left)**	**Picture 2 (upper right)**	**Picture 3 (bottom left)**	**Picture 4 (bottom right)**
**Practice**	egg	glass	child	mountain	**egg**
**Q1**	rain	butterfly	fire	**rain**	offering bowl
**Q2**	food	motorbike	shirt	flower	**banana**
**Q3**	insect	**ant**	goat	goose	tiger
**Q4**	swim	chicken	mosquito	**fish**	elephant
**Q5**	sting	**bee**	bird	cow	frog

**Q6**	horn	cat	**buffalo**	monkey	dog
**Q7**	hear	foot	arm	eye	**ear**
**Q8**	pray	school	house	**temple**	market
**Q9**	disease	**medicine**	fan	flag	bag
**Q10**	dream	climb	sit	walk	**sleep**
**Q11**	broom	wash	**clean**	bathe	wear
**Q12**	book	talk	**read**	eat	play

Note. The bold ones are the correct answers. The locations of the pictures on each sheet are indicated in parentheses. The pictures used in the task cannot be introduced here due to strict copyright restrictions.

Appendix 3

Questionnaire on Socialization Practices

Question	Please tick one of the five boxes according to how frequently you do the caregiving practice for your child.
**1** (D)	When my child doesn't obey a rule (e.g., He/She has not put his/her toys away; He/She has not completed a chore), then I punish him/her.
**2** (R)	I teach my child respect for the authorities.
**3** (HP)	When my child is defiant or oppositional, I give her/him a swat on the behind.
**4** (HP)	When my child gets on my nerves or is really exasperating, I occasionally resort to physical punishment (spanking, slapping).
**5** (PP)	When I see my child after a day of school, I make it possible to spend some time with him/her.
**6** (D)	When my child does something that I don't want him/her to do, I punish him/her.
**7** (AS)	I teach my child to make his/her own decisions.
**8** (PP)	In the evening, I talk with my child about the past and the coming day.
**9** (I)	When my child goes over the limit, I ignore her/him and don't speak to her/him for a long time (or, for a long enough time that s/he gets my point).
**10** (R)	I teach my child to be polite at school.
**11**(HP)	I spank my child when he/she is disobedient or naughty.
**12**(AS)	I teach my child to solve his/her own problems.
**13** (D)	When my child is impolite or behaves badly, I punish him/her.
**14** (PP)	I make time to listen to my child when he/she wants to tell me something.

**15** (I)	When my child does something that is not allowed, I only talk to him/her again when he/she behaves better.
**16** (PP)	When my child seems to have a problem, I discuss with him/her what is wrong.
**17** (I)	When my child does something that is not allowed, I don’t talk to him/her until he/she says sorry.
**18** (I)	When my child does something that is not allowed, I give him/her an angry look and I ignore him/her afterwards.
**19** (R)	I teach my child to obey rules.
**20** (R)	I teach my child to adapt to the habits in our family.
**21** (D)	When my child becomes too agitated or bothersome, I punish him/her.
**22** (D)	I punish my child when he/she makes a nuisance of himself/herself (e.g., because he/she nags, contradicts me, lies, argues).
**23** (PP)	I ask my child about his/her hobbies and interests.
**24** (R)	I teach my child to adapt to the rules at school.
**25** (AS)	I teach my child to be autonomous/independent when he/she’s outside of the house.
**26** (I)	When my child exaggerates things, I generally ignore what’s going on, then give her/him an insistent look.
**27 (HP)**	I slap my child when he/she has done something wrong.

Note. Items are from the original text of Meunier & Roskam (2009). The order of presentation was randomized by the authors. The symbols in parentheses attached to the item numbers represent the six domains, which were not included when distributed to respondents: AS: Autonomy Support; D: Discipline (Intrusive Control, in this study); HP: Harsh

Punishment; I: Ignoring; PP: Positive Parenting; R: Rules.

Appendix 4

Demographic, Socialization, and Task Homogeneity Verification Between Kindergartens

Variable	Private Kindergarten (N = 10) N	Private Kindergarten (N = 10) M (SD)	Public Kindergarten (N = 56) N	Public Kindergarten (N = 56) M (SD)	Analysis of Homogeneity: Mean difference	t	df	p	Hedges' g [95% CI]
Positive Parenting	10	4.12 (0.50)	56	3.71 (0.93)	0.41	2.03	22.19	.054	0.53 [−0.01, .06]
Rules	10	4.08 (0.71)	56	4.19 (0.72)	−0.11	−0.43	12.62	.672	−0.14 [−0.76, 0.49]
Harsh Punishment	10	2.20 (0.80)	56	2.07 (0.90)	0.13	0.48	13.41	.641	0.15 [−0.47, 0.76]
Ignoring	10	2.80 (0.97)	56	2.35 (0.97)	0.45	1.34	12.43	.204	0.43 [−0.23, 1.08]

Note. t = Welch's t-statistic. p-values and 95% CIs are unadjusted to preserve power for homogeneity detection. All six socialization domains were simultaneously analyzed to evaluate demographic homogeneity, with the two primary domains reported in the main text (see Table 1).

Appendix 5

Zero-order and Covariate-Adjusted Correlations between Age and Socialization Variables

Analysis Type	Socialization Variable	*N*	*r*	Corrected *p* (Holm)	95% CI
Zero-order (Pairwise)	Positive Parenting	66	.27	.182	[.03, .48]
	Rules	66	.10	.999	[−.14, .34]
	Intrusive Control	66	.04	.999	[−.20, .28]
	Harsh Punishment	66	−.11	.999	[−.34, .14]
	Ignoring	66	−.00	.999	[−.25, .24]
	Autonomy Support	66	.10	.999	[−.14, .34]
Partial (Language Controlled)	Positive Parenting	64	.11	.999	[−.14, .35]
	Rules	64	.07	.999	[−.18, .31]
	Intrusive Control	64	.02	.999	[−.23, .27]
	Harsh Punishment	64	−.10	.999	[−.34, .16]
	Ignoring	64	−.07	.999	[−.31, .18]
	Autonomy Support	64	.01	.999	[−.24, .26]

Note. All six domains were simultaneously included in the statistical analysis, with *p*-values adjusted for multiple comparisons across the family of six pairwise tests using the Holm method. 95% CIs were unadjusted.

Appendix 6

Zero-order and Covariate-Adjusted Correlations between Language and Socialization Variables

Analysis Type	Socialization Variable	*N*	*r*	Corrected *p* (Holm)	95% CI
Zero-order (Pairwise)	Positive Parenting	64	.36	.021	[.12, .56]
	Rules	64	.10	.999	[−.15, .34]
	Intrusive Control	64	.11	.999	[−.14, .35]
	Harsh Punishment	64	.01	.999	[−.24, .25]
	Ignoring	64	.01	.999	[−.24, .25]
	Autonomy Support	64	.21	.506	[−.04, .43]
Partial (Age Controlled)	Positive Parenting	64	.26	.233	[.01, .48]
	Rules	64	.05	.999	[−.20, .30]
	Intrusive Control	64	.08	.999	[−.17, .32]
	Harsh Punishment	64	.05	.999	[−.20, .29]
	Ignoring	64	.03	.999	[−.22, .28]
	Autonomy Support	64	.17	.965	[−.09, .40]

Note. All six domains were simultaneously included in the statistical analysis, with *p*-values adjusted for multiple comparisons across the family of six pairwise tests using the Holm method. 95 % CIs were unadjusted.

Appendix 7

Means and Standard Deviations of Caregiving Behaviors in Laos and Belgium, and the Effect Sizes

Context	Descriptive statistics	Positive Parenting	Rules	Harsh Punishment	Ignoring
Laos	*M*	3.77	4.17	2.09	2.42
(*N* =66)	*SD*	0.89	0.72	0.88	0.98
Belgium	*M*	4.15	4.50	2.24	2.01
(*N* = 348)	*SD*	0.49	0.45	0.81	0.84
Effect	*d*	−0.66	−0.66	−0.18	0.47
Sizes	95% CI	[−0.93, −0.40]	[−0.92, −0.39]	[−0.45, 0.08]	[0.21, 0.74]

Note. For Belgium, the maternal data from Roskam et al. (2014, p. 173, Table 1, T1) were cited. Responses are on a five-point Likert scale from never (1) to always (5). This table presents the descriptive statistics and effect sizes for the remaining four parenting domains, which were analyzed alongside the two primary domains reported in the main text (see Table 4) to ensure comprehensive data transparency. 95% CIs were unadjusted.

Appendix 8

Zero-order Correlation Analysis between EF Task Performance and Socialization Practices, as well as Regular and Bayesian Multiple Regression Analyses Predicting EF Task Performance

Analysis	Statistical summary	Positive Parenting	Rules	Harsh Punishment	Ignoring
Zero-order	*r*	.05	−.13	−.05	−.15
(*N* = 66)	p_{adj}	.999	.999	.999	.999
	95% CI	[−.19, .29]	[−.36, .11]	[−.29, .19]	[−.38, .09]
Multiple	*b* (*SE*)	−0.27 (0.10)	−0.29 (0.12)	−0.03 (0.10)	−0.19 (0.09)
Regression	p_{adj}	.060	.073	.999	.162
Analysis	95% CI	[−0.47, −0.07]	[−0.53, −0.06]	[−0.23, 0.17]	[−0.36, −0.01]
(*N* = 64)	*F*	.21.52	21.12	17.69	20.04
	R^2 (adj. R^2)	59 (.57)	.59 (.56)	.55 (.51)	.58 (.55)
Bayesian	Posterior	−0.25	−0.28	−0.03	−0.17
Multiple	Mean (*SD*)	(0.10)	(0.12)	(0.10)	(0.09)
Regression	BF_{10}	4.84	3.57	0.23	1.53
Analysis	95%	[−0.45, −0.05]	[−0.51, −0.05]	[−0.23, 0.17]	[−0.35, 0.00]
(*N* = 64)	Credible				
	Interval				

Note. EF = executive functions. This table presents statistics for the remaining four socialization domains. All six domains were analyzed as part of the same analytical family to maintain methodological rigor, while the two primary domains are reported in the main text (see Table 5). *p*-values for frequentist analyses (zero-order correlations and regression

coefficients) were adjusted using the Holm method across all six domains, while 95% CIs were unadjusted. In both regular and Bayesian multiple regressions, children's age, age squared, and language ability were controlled for. Bayesian statistics reflect unadjusted posterior distributions. For regular multiple regression analyses, overall model degrees of freedom are $df_{\text{regression}} = 4$ and $df_{\text{residual}} = 59$ (all overall models $p < .001$).

Appendix 9

Comprehensive Attrition Analysis for the Time-2 Eligible Sample: Baseline Demographic, Socialization, and Task Comparison between Time-1-only and Time-2 Participants

	Time-2 Participants		Time-1-only Participants		Attrition Analysis				
Variable	*N*	*M* (*SD*) or Count (%)	*N*	*M* (*SD*) or Count (%)	Mean difference	*t*	*df*	*p*	Hedges' *g* or Cramer's *V* [95% CI]
Positive Parenting	35	3.79 (0.82)	14	4.04 (0.86)	−0.25	0.95	23.10	52	−0.29 [−0.90, 0.32]
Rules	35	4.21 (0.60)	14	4.27 (0.93)	−0.06	0.22	17.53	25	−0.07 [−0.72, 0.57]
Harsh Punishment	35	1.97 (0.90)	14	2.21 (0.99)	−0.24	0.79	21.95	36	−0.25 [−0.86, 0.37]
Ignoring	35	2.27 (0.94)	14	2.63 (1.19)	−0.36	1.02	19.82	22	−0.32 [−0.95, 0.31]

Note. *t* = Welch's *t*-statistic. *p*-values and 95% CIs are unadjusted to preserve power for attrition bias detection. This table displays the remaining four socialization domains for the attrition analysis, while the demographics, tasks, and the two primary parenting domains are reported in the main text (see Table 6).

Appendix10

Details of the Mathematics Task

The test comprises 10 pages (20 × 28.5cm), with each page presenting one question with colored illustrations that were linked by a continuous story and culturally adapted from Laotian language textbooks. The experimenter could repeat questions when necessary, but was prohibited from paraphrasing or stressing certain words.

Contents of the Test

	Question	Answer	How to answer	Target skill	Note
**Q1**	Here are some children. How many children are there altogether?	two	orally or/and showing with fingers	subitizing or counting, cardinality	
**Q2**	They are going for a walk in the forest now. How many people are here altogether?	five	orally or/and showing with fingers	counting, cardinality	
**Q3**	On the way to the forest, there was a sign like this. What number is this?	three, seven	orally	numeral recognition	
**Q4**	They walked through a field. Are there more cows or more ducks here?	ducks	orally or/and pointing	magnitude comparison (discrete)	There are seven cows and nine ducks in the picture.
**Q5**	They arrived at the forest. This sign shows the number of animals in the forest. What number is this?	ten, twelve, seventeen	orally	numeral recognition	
**Q6**	Are there more elephants, more rabbits, or are they the same?	the same	orally or/and pointing	magnitude comparison (discrete; equality)	There are five rabbits and five elephants in the picture.
**Q7**	They found some bananas in the forest. Which one is bigger, the left one or the right one?	right	orally or/and pointing	magnitude comparison (continuous)	There are two identical bananas in different sizes in the picture.

**Q8**	There was a tree in the forest. Is the bird above the tree, or under the tree?	above	orally	spatial relations
**Q9**	There were five fish in the river. If one more fish joins them, how many fish will there be altogether?	six	orally or/and with fingers	simple addition
**Q10**	They are going back home now. There are five people here. If two of them get home first, how many people are left?	three	orally or/and with fingers	simple subtraction

Note. The pictures used in the task cannot be introduced here due to strict copyright restrictions. For Q3 & Q5, it was counted as correct only when all the numbers were answered.
